# Mortality and causes of death in patients with Sjögren disease in Western Australia: a population-based study

**DOI:** 10.1093/rheumatology/keag496

**Published:** 2026-09-10

**Authors:** Cristina Pelkas, Sue Lester, Fabien B Vincent, Catherine L Hill, Maureen Rischmueller, Johannes Nossent

**Affiliations:** College of Health, Adelaide University, Adelaide, South Australia, Australia; Rheumatology Department, The Queen Elizabeth Hospital, Adelaide, South Australia, Australia; Basil Hetzel Institute, The Queen Elizabeth Hospital, Adelaide, South Australia, Australia; College of Health, Adelaide University, Adelaide, South Australia, Australia; Rheumatology Department, The Queen Elizabeth Hospital, Adelaide, South Australia, Australia; Basil Hetzel Institute, The Queen Elizabeth Hospital, Adelaide, South Australia, Australia; Department of Medicine, Centre for Inflammatory Diseases, Monash University, Clayton, Victoria, Australia; College of Health, Adelaide University, Adelaide, South Australia, Australia; Rheumatology Department, The Queen Elizabeth Hospital, Adelaide, South Australia, Australia; Basil Hetzel Institute, The Queen Elizabeth Hospital, Adelaide, South Australia, Australia; College of Health, Adelaide University, Adelaide, South Australia, Australia; Rheumatology Department, The Queen Elizabeth Hospital, Adelaide, South Australia, Australia; Basil Hetzel Institute, The Queen Elizabeth Hospital, Adelaide, South Australia, Australia; Medical School, The University of Western Australia, Perth, Western Australia, Australia

**Keywords:** autoimmune disease, cardiovascular disease, infection, interstitial lung disease, lymphoma, mortality, Sjögren disease

## Abstract

**Objectives:**

Sjögren disease (SjD) is a systemic autoimmune disorder with emerging evidence showing increased mortality rates compared with the general population. We compared mortality risk and causes of death in Western Australian (WA) SjD patients with age/sex-matched controls and general population mortality rates.

**Methods:**

The WA Rheumatic Disease Epidemiological Registry (WARDER) was accessed in this data linkage study to identify patients with primary SjD, SjD associated with rheumatoid arthritis (SjD + RA) and SjD associated with other connective tissue disease (SjD + CTD) using ICD codes and age/sex matched controls (1:5) between 1980 and 2013. Mortality of SjD cohorts was compared with both age/sex-matched controls from WA and population mortality rates. Multiple cause of death and competing risk analysis was performed for SjD cohorts compared with controls.

**Results:**

A total of 3716 controls (1345 deaths) were compared with 457 primary SjD (204 deaths), 218 SjD + RA (149 deaths) and 77 SjD + CTD (38 deaths). Risk of mortality was increased in primary SjD [HR 1.8 (95% CI 1.5, 2.0)] and in associated SjD {SjD + RA: HR 2.1 [95% CI (1.8, 2.5)]; SjD + CTD: HR 3.1 (95% CI 2.2, 4.3)} compared with controls. Survival in primary SjD was 77% (95% CI 0.63, 0.94) at the end of follow-up (25 years). Mortality risk in primary and associated SjD was increased due to infection, ischaemic heart disease, lymphoid malignancy and interstitial lung disease.

**Conclusion:**

All-cause and specific-cause mortality was increased in primary and associated SjD compared with age/sex-matched controls in an Australian population.

## Graphical abstract

**Graphical Abstract:**
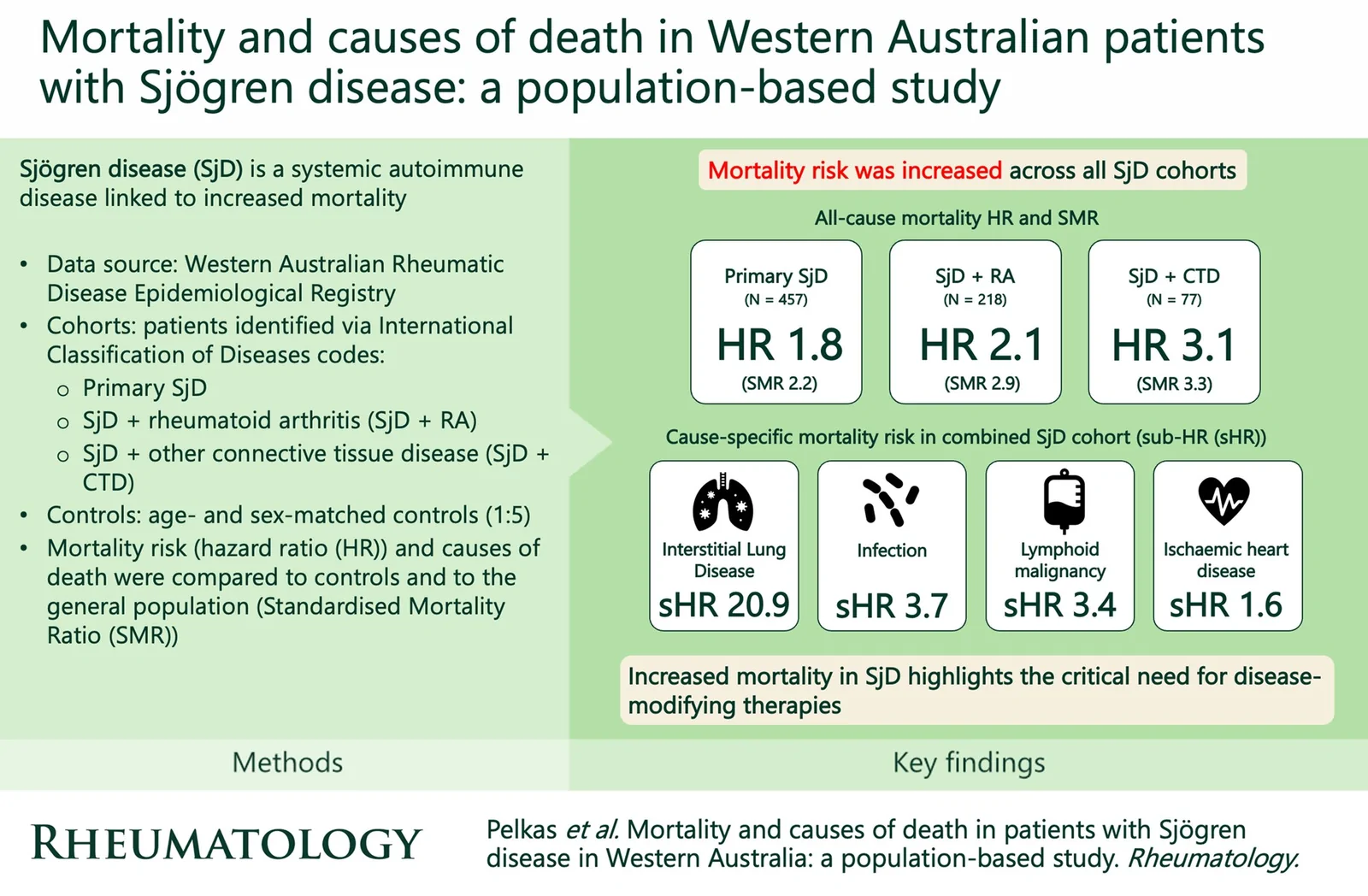

*Rheumatology* key messages

- All-cause mortality was increased in Western Australian patients with primary and associated SjD.
- Primary and associated SjD patients had increased mortality from infection, lymphoid malignancy, cardiovascular and respiratory disease when compared with controls.
- The increased mortality in SjD highlights the critical need for disease-modifying therapies.

## Introduction

Sjögren disease (SjD) is the second most common autoimmune rheumatic disease (AIRD), with international prevalence conservatively estimated between 0.01% and 0.05% (noting significant heterogeneity in this analysis) [1] and limited epidemiological data in the Australian context [2]. SjD affects exocrine glands causing dry eyes and mouth, in addition to serious systemic manifestations such as interstitial lung disease (ILD), lymphoma and vasculitis. SjD can be associated with other connective tissue diseases (CTD) (known as ‘associated SjD’) [3], occurring predominantly with rheumatoid arthritis (RA) (53%), systemic lupus erythematosus (SLE) (28%) and systemic sclerosis (13%) [4]. Since there are no approved treatments that prevent disease progression, SjD is managed empirically [5]. While increased lymphoma risk is well-characterized in SjD [6], emerging evidence suggests infection, cardiovascular and respiratory disease are also significant causes of mortality [7]. Knowledge regarding SjD mortality in Australia is lacking, including for Aboriginal and Torres Strait Islander peoples [8].

People with primary SjD have increased mortality rates compared with the general population. A large international cohort study by Brito-Zerón *et al.* (*N* = 11 372) demonstrated two-fold higher risk of all-cause and SjD-related death in patients with systemically active disease, with cardiovascular disease and infection causing almost 60% of deaths and malignancy causing 18% [9]. Similarly, a systematic review reported increased all-cause mortality in primary SjD {standardized mortality ratio (SMR) 1.46 [95% confidence interval (CI) 1.10, 1.93)]} compared with the general population [10]. However, data on mortality in associated SjD are limited and mortality is thought to be driven by the underlying AIRD [11, 12].

Between 5% and 10% of SjD patients develop non-Hodgkin lymphoma (NHL) [6] (up to 44-fold increased risk *vs* the general population [1, 13, 14]) which accounts for 30% of SjD-related deaths [7]. Evidence is conflicting regarding solid organ tumour risk in SjD, with a recent systematic review reporting only increased risk of thyroid cancer [relative risk (RR) 2.58; 95% CI 1.14–4.03], but not other solid organ malignancies [15]. Subsequently, a longitudinal cohort study reported elevated solid tumour risk in SjD patients [SIR 1.54 (95% CI 1.53, 1.55)] [16] and a prospective cohort study found that solid organ malignancies contributed to 14.4% of all deaths in SjD patients, primarily due to breast cancer, cholangiocarcinoma and hepatocarcinoma [7].

Given international evidence about increased mortality in SjD, the lack of Australian data regarding SjD mortality and limited, conflicting evidence surrounding causes of mortality in SjD patients compared with controls, we conducted this data-linkage study to investigate mortality risk and cause of death in SjD patients in Western Australia (WA).

## Methods

### Data sources

The WA Rheumatic Disease Epidemiological Registry (WARDER) contains population-level health data for patients requiring public or private hospital-level care for rheumatic diseases in WA (1980–2015). WARDER data is derived from the Hospital Morbidity Data Collection (HMDC; 1980–2015), Emergency Department Data Collection (EDDC; 2002–2015), WA Cancer Registry (1982–2015) and WA Births, Deaths and Marriages Registry (1980–2015) via the WA Data Linkage System (WADLS). International Classification of Diseases (ICD) Ninth Revision Clinical Modification (CM) (ICD-9-CM) was used until 1998, when it was replaced by the Tenth Revision Australian Modification (ICD-10-AM). The high linkage accuracy (99.7%) of WADLS enables longitudinal follow-up of individuals across multiple health datasets [17]. Aboriginal and/or Torres Strait Islander status was defined via self-report (in accordance with Australian Institute of Health and Welfare recommendations [18]).

### Cohort definitions

Patients with SjD were identified from hospital admission and emergency presentation data by ICD diagnosis codes [710.2 (ICD-9-CM) or M35.0 (ICD-10-AM)] and classified as either primary SjD (if no codes were identified for other rheumatological conditions) or associated SjD [if codes were present for RA (SjD + RA) or other CTD (SjD + CTD)] (see Supplementary Table S1). The date of the first ICD code for SjD was the patient index date, reflecting first capture rather than SjD onset in this prevalent cohort. Controls for each SjD patient (5:1 ratio), matched for birth year, sex and Aboriginal and/or Torres Strait Islander status, were selected from WADLS patients requiring hospital-level care (but with no relevant rheumatological diagnosis) and assigned the same index date as their matched SjD patient, then controls were subsequently pooled for analysis.

### Outcomes

The primary outcome was death and the secondary outcome was ICD-coded cause of death (COD) derived from the Medical Certificate of Cause of Death. COD consisted of both underlying (or direct) COD (which was only unambiguously assigned for the SjD cohort) and multiple causes of death data (MCoD). The underlying COD is ‘the disease or injury which initiated the train of morbid events leading directly to death’ [19]. The contributory COD are conditions that gave rise to the underlying COD, even with long intervals between the condition and death [19]. MCoD data incorporates underlying and contributory conditions, allowing more complete examination of all contributing COD (including chronic conditions and multimorbidity) on health outcomes [20].

Death linkage data was available between July 1980 and December 2014 for SjD patients and between November 1988 and December 2014 for controls. MCoD data were derived from record axis data, in which all information recorded on the death certificate is processed and captured as ICD codes. These data were available for the subset of deaths occurring between December 1996 and December 2013 in both SjD patients and controls, captured in up to 20 variables within the death linkage dataset.

A supplementary outcome was the number of comorbidities derived from the Elixhauser comorbidity index.

### Statistical analysis

Statistical analyses and visualization were performed using Stata software (v19.0, StataCorp LLC, College Station, TX, USA). Analysis primarily involved time-to-event (survival analysis) with both left censoring at the maximum of 15th birthday/linkage start date, and right censoring at the minimum of date of death/99th birthday/linkage end date. The analysis primarily compared primary SjD and all SjD cohorts combined with controls due to insufficient numbers of patients in associated SjD cohorts for reliable inference.

Demographic data at diagnosis for patients and controls were summarized using frequencies (percentages) or median and interquartile range (IQR).

#### Mortality and underlying COD in SjD compared with population mortality rates: SMR and relative survival

Expected values for relative survival and SMR calculations utilized age, sex and calendar year-matched WA population mortality rates (available from the Australian Bureau of Statistics Data Explorer Datasets [21]) and for underlying COD utilized Australian age, sex and calendar year-matched cause-specific mortality rates from the General Record of Incidence of Mortality (GRIM) books [22].

Marginal relative survival (the ratio of observed to expected survival) was estimated using the Stata ado ‘stpp’ [23] which estimates the relative survival function at each death time. SMR is the ratio of the cumulative observed deaths to cumulative expected deaths. Expected deaths were estimated by a life-table approach (1-year intervals) using the Stata ado ‘strs’ [24] adopting the Ederer II method [25] and SMR calculated by Poisson regression (with a robust variance-covariance matrix). Results were age-standardized by weighting to the Australian 2001 standard population (age groups 15–44, 45–54, 55–64, 65–74, 75+ years) ensuring comparability between results for the different cohorts.

SMR were calculated for both overall mortality and underlying COD. The ICD codes used for analysis of underlying COD are reported in Supplementary Table S2.

#### Mortality and MCoD analysis in SjD compared with controls

Survival models for mortality in SjD compared with controls utilized age as the analysis time scale, with delayed entry to accommodate age differences at time of entry into the analysis. Overall mortality in SjD cohorts was compared with control mortality rates by Cox regression, with hazard ratios (HR) estimated under the proportional hazards assumption. Time-varying effects (violation of the proportional hazards assumption) were investigated using a Royston-Parmar model, estimated using the Stata ado ‘stpm3’ [26] utilizing splines to model the time scale. The best fitting model, as judged by the Akaike information criteria, was equivalent to a Weibull model, df [1], with time-varying effects, also df [1], for each participant subgroup.

Classification of specific COD listed in MCoD data was based around the hierarchical structure of major ICD chapters (with the addition of dementia, diabetes, renal disease and liver disease). The ICD codes utilized are reported in Supplementary Table S2.

Analysis utilized a competing risk approach (Fine-Gray Model) with individual deaths classified as either including the COD of interest or not (a competing event). This enables estimation of the cumulative incidence function (CIF), the probability that an individual COD contributed to death. Results are reported as subHazard Ratios (sHR), which measure the effect of covariates on the CIF, for both primary SjD and all SjD compared with controls.

#### Comorbidity burden

The Elixhauser Comorbidity Index is a validated method for identifying medical conditions (such as malignancy, heart failure, diabetes, alcohol use and obesity) using ICD codes to predict key patient outcomes (e.g. in-hospital mortality) [27]. Comorbidities were assessed at each hospital admission utilizing hospital discharge diagnosis information, which included up to 22 ICD-coded diagnosis variables: a principal diagnosis, co-diagnosis and 20 additional diagnosis variables.

Participants were included if they had at least one hospital event after their index date. Individual comorbidities were assessed cumulatively during follow-up, using available hospital admission data, with the Stata ado elixhauser [28] which utilizes the Quan *et al.* [29] ICD coding for 31 comorbidities. The total number of comorbidities for each individual by the last hospital admission (excluding the ‘Rheumatoid Arthritis, Collagen/Vascular Disease’ component and collapsing over diabetes and hypertension components) was analysed between patient subgroups by negative binomial regression, adjusted for sex and age (at last hospital admission) classified into five groups (20–44, 45–54, 55–64, 65–74, 75+ years old) with reporting of marginal population-averaged means.

Mortality in SjD patient cohorts *vs* matched controls was compared using Cox proportional hazards regression. In a sensitivity analysis, mortality HRs were estimated across three models: unadjusted (Model 1) and adjusted for covariates sex (Model 2) and sex plus Elixhauser comorbidities (Model 3).

### Ethics approval

The project was approved by the Human Research Ethics Committee at the WA Department of Health (HREC # 2016.24).

## Results

### Cohort demographic characteristics

The study population characteristics are shown in Table 1. The analysis included 3716 controls (1345 deaths) over a maximum of 25 years of follow-up and 457 primary SjD (204 deaths), 218 SjD + RA (149 deaths) and 77 SjD + CTD (38 deaths) patients, with a total of 752 SjD patients (391 deaths) over a maximum of 33 years of follow-up. The cohorts were predominantly female [controls: 87% (*N* = 3246); primary SjD: 86% (*N* = 391); SjD + RA: 89% (*N* = 195); SjD + CTD 92% (*N* = 71), respectively]. The SjD + CTD patients were approximately a decade younger at their index date {median (IQR) age: [54 (44, 70) years] compared with pooled controls [63 (51, 73) years], primary SjD [64 (50, 75) years] and SjD + RA [65 (56, 72) years]}. Aboriginal and/or Torres Strait Islander peoples comprised 2% (*N* = 14) of the primary and associated SjD cohort and 2% (*N* = 69) of controls. The mean number of cumulative Elixhauser comorbidities (age and sex adjusted) in 3190 controls and 746 SjD patients was higher in primary SjD (3.8, 95% CI 3.4, 4.1) than controls (1.9, 95% CI 1.8, 2.0).

**Table 1:** Sjögren disease and control cohort characteristics.

	Controls (N = 3716)	Primary SjD (N = 457)	SjD + RA (N = 218)	SjD + CTD (N = 77)
Deaths*, n*	1345	204	149	38
Follow-up (person-years)	46736.4	4527.5	2541.1	947.0
Females*, n (%)*	3246 (87%)	391 (86%)	195 (89%)	71 (92%)
Index age (years), median (IQR)	63 (51, 73)	64 (50, 75)	65 (56, 72)	54 (44, 70)
Aboriginal and/or Torres Strait Islander*, n (%)*	69 (2%)	7 (2%)	≤5	≤5
Elixhauser comorbidities (95% CI)^a^	1.9 (1.8, 2.0)	3.8 (3.4, 4.1)	4.7 (4.2, 5.3)	6.0 (4.8, 7.3)

a The mean cumulative number of Elixhauser comorbidities by the final hospital admission, adjusted for age and sex.

CI: confidence interval; CTD: connective tissue disease; IQR: interquartile range; RA: rheumatoid arthritis; SjD: Sjögren disease.

### Mortality risk in SjD

We first evaluated all-cause mortality in SjD compared with WA population mortality rates, using relative survival (Fig. 1) and SMR (Table 2). Relative survival was decreased in all SjD cohorts [primary SjD: 0.77 (95% CI 0.63, 0.94), SjD + RA: 0.68 (95% CI 0.55, 0.85), SjD + CTD: 0.57 (95% CI 0.36, 0.91)] compared with WA population mortality rates (Supplementary Table S3), which was reflected in SMR significantly >1. Of note, all-cause mortality in our age/sex-matched controls was comparable to WA population rates [relative survival at 25 years 0.99 (95% CI 0.95, 1.04)].

**Figure 1:**
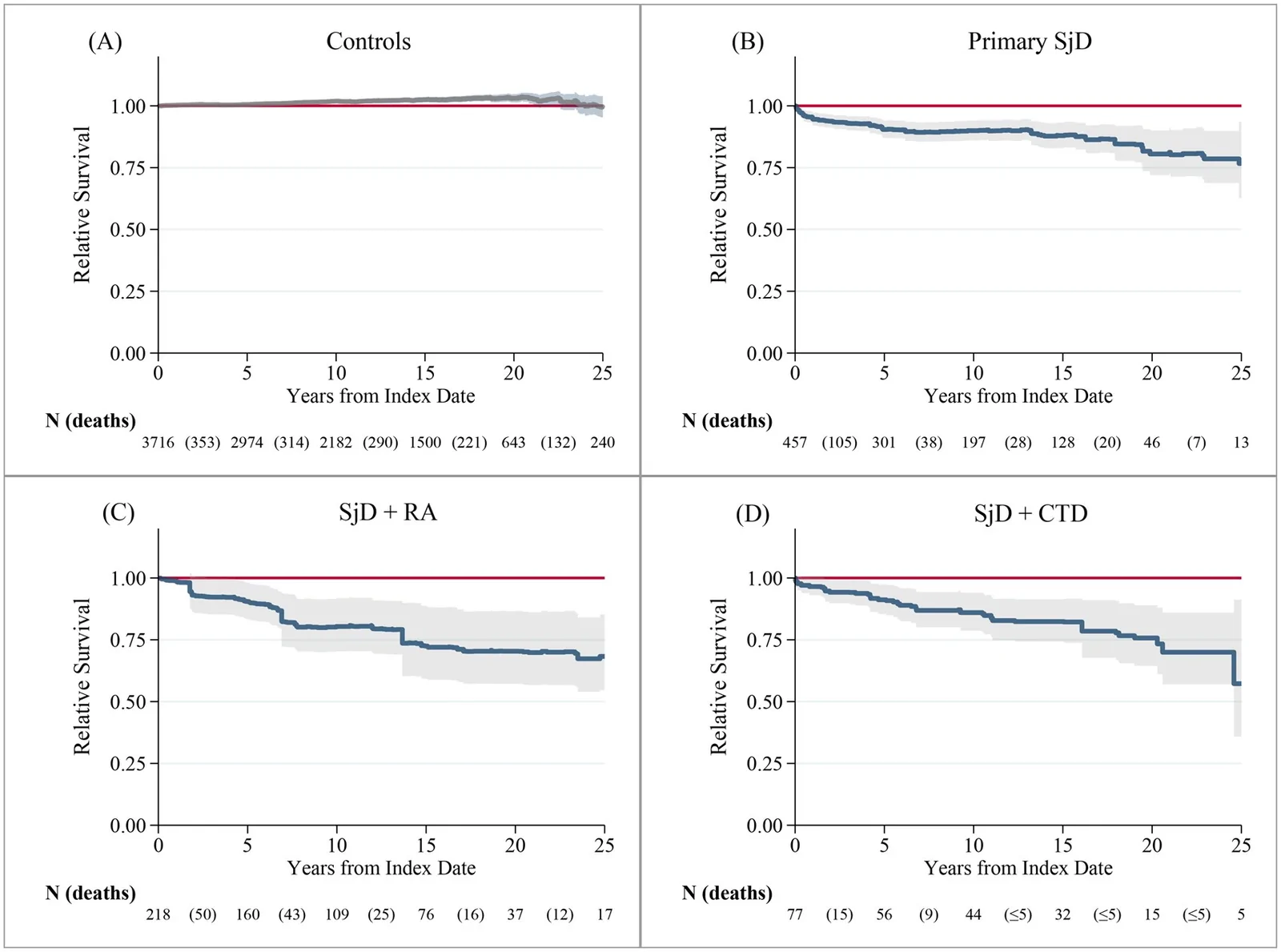
Marginal Relative Survival for Sjögren disease cohorts and controls compared with all-cause population mortality rates. Shaded areas represent 95% confidence intervals. (**A**) Marginal relative survival in age- and sex-matched controls compared to population mortality rates. (**B**) Marginal relative survival in primary SjD compared to population mortality rates. (**C**) Marginal relative survival in SjD + RA compared to population mortality rates. (**D**) Marginal relative survival in SjD + CTD compared to population mortality rates. CTD: connective tissue disease; RA: rheumatoid arthritis; SjD: Sjögren disease

**Table 2:** Standardized mortality ratios and mortality hazards ratios for Sjögren disease cohorts.

Cohorts	SMR (95% CI)^a^	*P*-value	HR (95% CI)^b^	*P*-value
Controls	0.9 (0.9, 1.01)	0.10	1	–
Primary SjD	2.2 (1.8, 2.6)	<0.001	1.8 (1.5, 2.0)	**<0.001**
SjD + RA	2.9 (2.3, 3.6)	<0.001	2.1 (1.8, 2.5)	**<0.001**
SjD + CTD	3.3 (2.2, 4.9)	<0.001	3.1 (2.2, 4.3)	**<0.001**
All SjD combined	2.8 (1.7, 4.4)	<0.001	2.0 (1.8, 2.2)	**<0.001**

a All-cause standardized mortality ratios for Sjögren disease cohorts compared with Western Australian population mortality rates at 25 years of follow-up.

b All-cause mortality hazards ratios for Sjögren disease cohorts compared with age- and sex-matched controls.

CI: confidence interval; CTD: connective tissue disease; RA: rheumatoid arthritis; SjD: Sjögren disease; SMR: standardized mortality ratio. Bold text indicates significant *P*-values.

All-cause mortality HR were increased in each SjD cohort compared with controls (Table 2), reflecting the same trends as SMR. The increased mortality for all SjD compared with controls was HR 2.0 (95% CI 1.8, 2.2). However, there was evidence of violation of the proportional hazards assumption (i.e. HR were not constant over the period of follow-up) for each SjD group (*P < *0.001), suggesting that mortality risk varied over time. Adjustment for sex had minimal effect on the mortality HR, as SjD cohorts and controls were well matched for sex; however, additional adjustment for the covariate Elixhauser comorbidity burden resulted in modest attenuation of the mortality HR in the SjD cohorts, which remained significant (Supplementary Table S4).

The hazard rates for each participant subgroup, allowing for non-proportional hazards, is shown in Supplementary Fig. S1A. The time-varying mortality hazard rates increased substantially from 60 years of age for SjD cohorts compared with controls. Supplementary Fig. S1B demonstrates that the time-varying effect is a modest increase in HR with increasing age.

### Underlying COD in SjD

We next compared underlying COD in primary and combined SjD compared with Australian population cause-specific mortality rates. The most frequent underlying COD were circulatory diseases, neoplasms, respiratory disease and musculoskeletal diseases, collectively accounting for ∼75% (294/391) of deaths, all increased in SjD compared with Australian population cause-specific mortality rates (Table 3). Although less frequent (*N* = 10), death due to infection was also increased in the combined SjD cohort.

**Table 3:** Underlying cause of death: standardized mortality ratios in Sjögren disease patients compared with Australian population cause-specific mortality rates.

GRIM category	Primary SjD			All SjD combined		
	Deaths, *n*	SMR (95% CI)	*P*-value	Deaths, *n*	SMR (95% CI)^a^	*P*-value
Circulatory diseases	73	1.4 (1.2 1.8)	**0.001**	131	1.7 (1.3, 2.1)	**< 0.001**
Coronary heart disease	40	1.6 (1.1, 2.2)	**0.007**	69	1.9 (1.4, 2.5)	**<0.001**
Cerebrovascular disease	10	0.8 (0.3, 2.0)	0.56	16	0.9 (0.5, 1.5)	0.61
Neoplasms	43	2.3 (1.2, 4.6)	**0.02**	66	1.9 (1.1, 3.4)	**0.03**
Lung cancer	9	2.9 (0.9, 9.1)	0.06	15	3.0 (1.3, 7.0)	**0.01**
Respiratory diseases	22	3.5 (1.7, 7.2)	**<0.001**	47	4.2 (2.7, 6.5)	**<0.001**
Infections	6	–	–	10	7.1 (2.5, 19.9)	**<0.001**

a Age standardized to the Australian 2001 Standard Population.

CI: confidence interval; GRIM: General Record of Incidence of Mortality books; SjD: Sjogren disease; SMR: standardized mortality ratio. Bold text indicates significant *P*-values.

### MCoD in SjD

We then examined MCoD for primary SjD and combined SjD compared with age- and sex-matched controls. All-cause mortality sHR for this data subset were substantively the same as those reported in Table 2 for the full dataset (data not shown). Findings from the MCoD analysis (Table 4) were broadly similar to the underlying COD analysis for the full cohort (Table 3), but enabled a more nuanced analysis of specific causes in the presence of controls.

**Table 4:** Multiple cause of death analysis in patients with Sjögren disease compared with controls.

*Cause of death*	*Group*	*Deaths, n*	*sHR (95% CI)*	*P-value*
*Chapter I: **Certain Infectious and Parasitic Diseases***	Controls	57	1	–
	Primary SjD	17	2.8 (1.6, 4.8)	**<0.001**
	All SjD	38	3.7 (2.5, 5.6)	**<0.001**
*Chapter II: **Neoplasms***	Controls	269	1	–
	Primary SjD	41	1.4 (1.0, 2.0)	**0.04**
	All SjD	62	1.3 (0.9, 1.7)	0.13
*Solid organ malignant neoplasms*	Controls	254	1	–
	Primary SjD	35	1.3 (0.9, 1.8)	0.20
	All SjD	53	1.1 (0.8, 1.5)	0.48
*Lung cancer*	Controls	45	1	–
	Primary SjD	7	–	–
	All SjD	10	1.2 (0.6, 2.4)	0.63
*Malignant neoplasms of lymphoid, hematopoietic and related tissue*	Controls	19	1	–
	Primary SjD	10	4.7 (2.2, 10.0)	**< 0.001**
	All SjD	12	3.4 (1.6, 6.9)	**0.001**
*Chapter IX: **Diseases of the Circulatory System***	Controls	564	1	–
	Primary SjD	89	1.6 (1.3, 2.0)	**<0.001**
	All SjD	147	1.6 (1.3, 1.9)	**<0.001**
*Coronary heart disease*	Controls	246	1	–
	Primary SjD	47	1.9 (1.4, 2.6)	**<0.001**
	All SjD	69	1.6 (1.2, 2.1)	**0.001**
*Other (cardiomyopathies, dysrhythmias, etc)*	Controls	*153*	1	–
	Primary SjD	21	1.3 (0.8, 1.98)	0.35
	All SjD	35	1.2 (0.9, 1.78)	0.28
*Hypertensive disease*	Controls	131	1	–
	Primary SjD	17	1.2 (0.7, 1.98)	0.76
	All SjD	31	1.2 (0.9, 1.90)	0.23
*Cerebrovascular disease*	Controls	*146*	*1*	–
	Primary SjD	18	1.1 (0.7, 1.85)	0.66
	All SjD	27	1.0 (0.7, 1.50)	0.96
*Heart failure*	Controls	*149*	*1*	–
	Primary SjD	10	0.6 (0.3, 1.10)	0.09
	All SjD	22	0.8 (0.5, 1.2)	0.26
*Pulmonary circulation disorders*	Controls	*13*	*1*	–
	Primary SjD	≤5	–	–
	All SjD	13	5.4 (2.5, 11.6)	**<0.001**
*Chapter X: **Respiratory Diseases***	Controls	303	1	–
	Primary SjD	57	1.8 (1.4, 2.5)	**<0.001**
	All SjD	108	2.2 (1.7, 2.7)	**<0.001**
*Respiratory infections*	Controls	955	1	–
	Primary SjD	158	1.9 (0.6, 5.4)	0.26
	All SjD	266	2.2 (1.0, 5.1)	0.054
*Obstructive pulmonary disease*	Controls	*89*	1	–
	Primary SjD	17	1.8 (1.0, 3.0)	**0.03**
	All SjD	31	1.9 (1.3, 2.9)	**0.002**
*Bronchiectasis*	Controls	≤5	–	–
	Primary SjD	6	–	–
	All SjD	13	14.1 (5.1, 39.2)	**<0.001**
*Interstitial lung disease*	Controls	≤5	≤5	–
	Primary SjD	9	20.4 (6.7, 61.8)	**<0.001**
	All SjD	15	20.9 (6.4, 61.8)	**<0.001**
*Chapter III: **Diseases of the blood and blood-forming organs & certain disorders involving the immune mechanism***	Controls	23	–	–
	Primary SjD	8	3.2 (1.4, 7.1)	**0.006**
	All SjD	15	3.5 (1.8, 6.7)	**<0.001**
***Other: Dementia***	Controls	200	1	–
	Primary SjD	20	0.9 (0.6, 1.4)	0.55
	All SjD	31	0.8 (0.6, 1.2)	0.26
***Other: Diabetes***	Controls	76	1	–
	Primary SjD	16	2.0 (1.1, 3.4)	**0.015**
	All SjD	21	1.5 (0.9, 2.4)	0.11
***Other: Renal disease***	Controls	69	1	–
	Primary SjD	14	1.8 (1.0, 3.3)	**0.038**
	All SjD	18	1.4 (0.8, 2.3)	0.21
***Other: Liver disease***	Controls	10	1	–
	Primary SjD	8	–	–
	All SjD	10	5.3 (2.2, 12.6)	**<0.001**
***Rheumatological disease*** ^a^	Controls	≤5	–	–
	Primary SjD	22	–	–
	All SjD	66	–	–
***Sjögren disease*** ^b^	Controls	≤5	–	–
	Primary SjD	15	–	–
	All SjD	19	–	–

a No comparative analysis performed as insufficient control numbers. Rheumatological disease incorporates the ICD codes listed in Supplementary Table S2, including connective tissue diseases, rheumatoid arthritis, polymyalgia rheumatica and vasculitis.

b No comparative analysis performed as insufficient control numbers.

CI: confidence interval; subHR: subhazard ratio; SjD: Sjögren disease. Bold text indicates significant *P*-values.

Analysis was restricted to deaths which occurred between December 1996 and December 2013 when MCoD data was available. Analysis numbers consisted of 3427 controls (955 deaths), 415 primary SjD (158 deaths), 156 SjD + RA (81 deaths) and 64 SjD + CTD (27 deaths). Results are reported as subHazard Ratios (sHR) from a competing risks analysis with death due to other causes the competing event.

Combined SjD cohorts showed significantly increased mortality from infections [sHR 3.7 (95% CI 2.5, 5.6)] and circulatory disease [sHR 1.6 (95% CI 1.3, 1.9)] including ischaemic heart disease [sHR 1.6 (95% CI 1.2, 2.1)] and pulmonary circulation disorders [sHR 5.4 (95% CI 2.5, 11.6)] compared with age/sex-matched controls. Increased respiratory deaths [sHR 2.2 (95% CI 1.7, 2.7)] in combined SjD cohorts were due predominantly to ILD [sHR 20.9 (95% CI 6.4, 61.8)] and obstructive pulmonary disease [sHR 1.9 (95% CI 1.3, 2.9)] [including bronchiectasis (sHR 14.1 (95% CI 5.1, 39.2)] compared with controls, but there was no significant increase in mortality due to respiratory infections.

While mortality was increased in combined SjD cohorts from lymphoid neoplasms [sHR 3.4 (95% CI 1.6, 6.9)] compared with age- and sex-matched controls, death due to neoplasms overall [sHR 1.3 (95% CI 0.9, 1.7), *P* = 0.13] and solid organ malignancies [sHR 1.1 (95% CI 0.8, 1.5), *P* = 0.48] were not significantly elevated. Cross-referencing with International Classification of Diseases for Oncology (ICD-O) codes in cancer linkage data showed that the majority of haematological cancers in SjD were NHL/B cell lymphomas (ICD-O 9590–9729, *N* = 8). MCoD analysis did not confirm the association with lung cancer deaths observed in the underlying COD analysis (Table 3). The CIF for the most frequently identified major ICD chapter COD in SjD (cardiovascular diseases, respiratory diseases, neoplasms and infections) compared with controls is shown in Fig. 2, with increased cumulative incidence of these COD throughout the lifespan in SjD cohorts compared with controls, consistent with time-varying mortality patterns described earlier. In addition, the CIF represents the probability that a death with a specific cause has occurred by a given age, with the CIF over a lifespan providing an estimate of the average proportion of SjD patients who have died with these specific causes.

**Figure 2:**
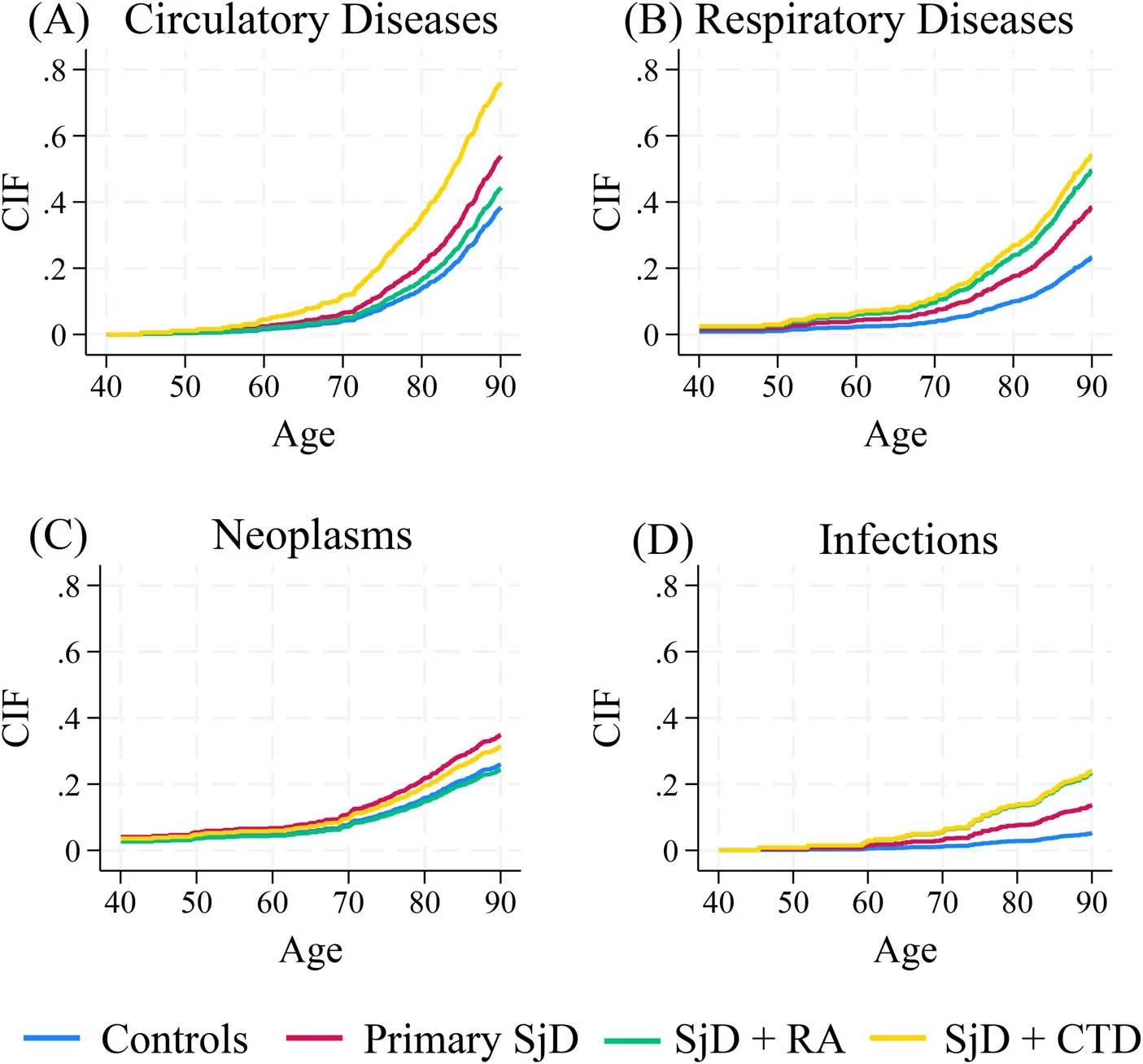
Cumulative Incidence Function (by age) for the four major International Classification of Diseases Codes chapter classifications most frequently contributing to mortality in Sjögren disease patients and controls (multiple cause of death analysis). (**A**) Cumulative incidence function (CIF) of circulatory diseases in SjD cohorts by age. (**B**) CIF of respiratory diseases in SjD cohorts by age. (**C**) CIF of neoplasms in SjD cohorts by age. (**D**) CIF of infections in SjD cohorts by age. CTD: connective tissue disease; CIF: cumulative incidence function; RA: rheumatoid arthritis; SjD: Sjögren disease

## Discussion

Significant knowledge gaps exist regarding risk and causes of mortality in primary and associated SjD in Australia. We found all-cause mortality risk was increased in SjD patients compared with WA population mortality rates. The increased all-cause mortality risk that we observed in primary SjD patients [SMR 2.2 (95% CI 1.8, 2.6)] compared with WA population mortality rates is comparable to findings of larger international cohorts [SMR 1.46 (95% CI 1.10–1.93)] [10]. Higher all-cause risk of mortality was also observed in SjD + CTD [SMR 3.3 (95% CI 2.2, 4.9)] and SjD + RA [SMR 2.9 (95% CI 2.3, 3.6)], consistent with international reports of higher all-cause mortality risk in the associated AIRD {SLE [SMR 2.66 (95% CI 2.09, 3.39)] [30], systemic sclerosis [SMR 2.72 (95% CI 1.93, 3.83)] [31] and RA [SMR 1.59 (95% CI 1.34, 1.70)] [32]} and previous WA data [33–35]. Our findings are generally consistent with previously published mortality risk for primary SjD and the associated CTD. All-cause mortality HR were also increased in each SjD cohort compared with age- and sex-matched controls, reflecting the same trends as population SMR.

Marginal relative survival was decreased in all SjD cohorts compared with WA population mortality rates, most significantly for the SjD + CTD cohort. Importantly, the marginal relative survival of the age- and sex-matched control population was comparable to WA population mortality rates at 25 years, demonstrating that survival rates of age- and sex-matched controls were representative of the WA population.

Our MCoD analysis indicated that circulatory disease was an important contributor to mortality in SjD patients [sHR 1.6 (95% CI 1.3, 1.9)], with significantly increased mortality risk due to ischaemic heart disease and pulmonary circulation disorders (including pulmonary embolism, primary and secondary pulmonary hypertension) compared with matched controls. A recent systematic review found no significant association between SjD and cardiovascular mortality [RR 1.48 (95% CI 0.77, 2.85), *P* = 0.24], despite SjD patients having higher risk for cardiovascular morbidity such as coronary artery disease [RR 1.34 (95% CI 1.06, 1.38), *P* = 0.01] *vs* control populations [36]. We did not have information surrounding other cardiovascular risk factors in our cohort (e.g. smoking rates), which may have contributed to our finding of elevated cardiovascular mortality.

Respiratory disease contributed significantly to mortality in our SjD cohorts, especially ILD [sHR 20.9 (95% CI 6.4, 61.8)], bronchiectasis [sHR 14.1 (95% CI 5.1, 39.2)] and obstructive pulmonary disease [sHR 1.9 (95% CI 1.3, 2.9)], underlining the importance of identification and management of respiratory manifestations in SjD [37]. Patients with SjD-related ILD by definition have systemic disease activity [37], known to be associated with increased mortality [9].

Elevated mortality risk due to lymphoid neoplasms in our cohort compared with matched controls [sHR 3.4 (95% CI 1.6, 6.9)] is supported by previous literature [7] and also reflects systemically active SjD. We found conflicting evidence of mortality from solid organ malignancy in SjD patients, with increased risk of mortality from lung cancer in SjD patients compared with Australian population cause-specific mortality rates [SMR 3.0 (95% CI 1.7, 5.2)], but no significantly increased risk of mortality from solid organ malignancies overall in SjD patients [sHR 1.1 (95% CI 0.8, 1.5), *P* = 0.48] compared with age- and sex-matched controls. Multiple studies have shown elevated solid tumour risk in SjD [7, 16, 38] with malignancy contributing to a significant proportion of overall deaths [7, 9]. Our conflicting findings about solid organ malignancy may be due to small numbers of deaths from malignancy (*N* = 46), a relatively short follow-up window of 25 years and publicly funded access to malignancy screening and treatment in Australia for citizens and permanent residents.

Mortality risk due to infection was significantly increased in the combined SjD cohort compared with age- and sex-matched controls. The importance of infection as a COD in SjD has been shown consistently in multiple studies [7, 9, 39]. In addition to underlying immune dysfunction in AIRD, serious infection risk is elevated with chronic glucocorticoid treatment used in severe AIRD and SjD complications such as ILD [40].

The predominance of female patients in our study is consistent with the known higher ratio of female *vs* male SjD patient demographic (9:1) [4]. The higher burden of Elixhauser comorbidities in SjD cohorts may contribute to increased mortality, with further research needed to determine whether these comorbidities are manifestations of SjD, reflect a shared predisposition or result from adverse effects of treatments. To our knowledge, this is the first study of SjD mortality that identified self-reported Aboriginal and Torres Strait Islander ethnicity in primary SjD patients [8], although subgroup analysis of COD was not possible due to small numbers (*N* = 14). Reports indicate that in Aboriginal and Torres Strait Islander peoples, SLE mortality rates are six times higher [41] and RA mortality rates are three times higher than the general Australian population [42], revealing the critical need for improved research and models of care.

This study comprises the most comprehensive analysis of mortality risk and causes in SjD using both curated ICD-coded COD information and a matched control population, in addition to population mortality rates, which alone are used in most studies of mortality in SjD [7, 10, 36, 43]. Limitations include absence of information regarding the SjD diagnostic criteria used, diagnosis date, disease duration (with inclusion of patients at different stages of disease), measures of disease activity, ethnicity, smoking status and socio-economic status, which may have influenced mortality outcomes [44] and generalizability of results. Patients were drawn from a hospital-based cohort with potentially more severe SjD and/or comorbidities than patients not needing hospitalization. Furthermore, while controls were drawn from a hospitalized cohort and may differ from the general population (especially with respect to comorbidity burdens), we demonstrated mortality outcomes (e.g. relative survival) in controls that are commensurate with WA population data. Additional limitations include the absence of baseline comparison between SjD and control cohorts for disease composition, which may have led to unbalanced confounding factors and potential bias in mortality comparison. To partially address this, a sensitivity analysis was performed of the mortality HRs adjusted for cumulative comorbidities, with modest reduction of HRs, which remained significant. As data linkage of the cohort was only until 2013, outcomes may not reflect contemporary therapies for the AIRD associated with SjD (noting there are no proven disease-modifying treatments for SjD) and improvements in therapies and multidisciplinary care for SjD complications such as ILD. Finally, COD classification based on death certificates may be incomplete, due to reliance on medical professionals’ subjective judgment.

In conclusion, primary and associated SjD were associated with significantly increased all-cause mortality and mortality due to infection, lymphoid malignancy, cardiovascular and respiratory disease compared with age- and sex-matched controls in an Australian population. As systemic disease activity predicts mortality in SjD [9], this study confirming elevated mortality risk of Australian SjD patients highlights the importance of assiduous clinical monitoring and management of systemic disease activity, and the critical need for accelerated development of disease-modifying therapies for SjD. The significant contribution of cardiovascular disease and infection to mortality necessitates early cardiovascular risk factor management and vigilance for preventing and treating infections to improve overall survival. Further research is warranted to explore underlying mechanisms leading to increased cardiovascular morbidity and mortality in SjD, as well as impacts of socio-economic status, geographic location, comorbidities, and ethnicity on SjD mortality in Australia.

## Supplementary Material

keag496_Supplementary_Data

## Data Availability

The data underlying this article were provided by the Western Australia Data Linkage System. The data cannot be shared publicly to minimize reidentification of individuals. Data will be shared on request to the corresponding author with permission of the Western Australia Data Linkage System.

## References

[1] Beydon M , McCoy S, Nguyen Y et al Epidemiology of Sjögren syndrome. Nat Rev Rheumatol 2024;20:158–69. 10.1038/s41584-023-01057-6 38110617

[2] Roper L , Cameron E, Yu T et al Prevalence of vasculitis, systemic lupus erythematosus, rheumatoid arthritis, systemic sclerosis, idiopathic inflammatory myopathies and spondyloarthritis in Australia: a systematic review and meta-analysis. Intern Med J 2025;55:1985–96. 10.1111/imj.70210 PMC12704404 40955101

[3] Baldini C , Bartoloni E, Bombardieri M. Highlights of the 15th international symposiumon Sjögren’s syndrome. Clin Exp Rheumatol 2022;40:2201–10. 10.55563/clinexprheumatol/yt6g9t 36541239

[4] Seror R , Chiche L, Beydon M et al Estimated prevalence, incidence and healthcare costs of Sjögren’s syndrome in France: a national claims-based study. RMD Open 2024;10:1–9. 10.1136/rmdopen-2023-003591 PMC10840052 38307699

[5] Pelkas C , Franke KB, Vincent FB, Rischmueller M. Novel therapies in Sjögren’s disease: a systematic review of the literature. Best Pract Res Clin Rheumatol 2025;39:102084. 10.1016/j.berh.2025.102084 40628622

[6] Nocturne G , Pontarini E, Bombardieri M, Mariette X. Lymphomas complicating primary Sjögren’s syndrome: from autoimmunity to lymphoma. Rheumatology (Oxford) 2021;60:3513–21. 10.1093/rheumatology/kez052 PMC8328496 30838413

[7] Lovgach OR , Plaza Z, Castro MF et al Mortality in patients with Sjögren disease: a prospective cohort study identifying key predictors. J Rheumatol 2025;52:jrheum.2024–262. 10.3899/jrheum.2024-1033 39814434

[8] Eades LE , Sines J, Hoi AY et al Autoimmune rheumatic disease in Australian Aboriginal and Torres Strait Islander peoples: what do we know? Semin Arthritis Rheum 2024;65:152354. 10.1016/j.semarthrit.2023.152354 38237231

[9] Brito-Zerón P , Flores-Chávez A, Horváth IF et al; Sjögren Big Data Consortium. Mortality risk factors in primary Sjögren syndrome: a real-world, retrospective, cohort study. EClinicalMedicine 2023;61:102062. 10.1016/j.eclinm.2023.102062 PMC10344811 37457113

[10] Huang H , Xie W, Geng Y, Fan Y, Zhang Z. Mortality in patients with primary Sjögren’s syndrome: a systematic review and meta-analysis. Rheumatology (Oxford) 2021;60:4029–38. 10.1093/rheumatology/keab364 33878179

[11] Nossent JC , Swaak AJ. Systemic lupus erythematosus VII: frequency and impact of secondary Sjøgren’s syndrome. Lupus 1998;7:231–4. 10.1191/096120398678920046 9643312

[12] Scherlinger M , Lutz J, Galli G et al Systemic sclerosis overlap and non-overlap syndromes share clinical characteristics but differ in prognosis and treatments. Semin Arthritis Rheum 2021;51:36–42. 10.1016/j.semarthrit.2020.10.009 33360228

[13] Fallah M , Liu X, Ji J et al Autoimmune diseases associated with non-Hodgkin lymphoma: a nationwide cohort study. Ann Oncol 2014;25:2025–30. 10.1093/annonc/mdu365 25081899

[14] Kassan SS , Thomas TL, Moutsopoulos HM et al Increased risk of lymphoma in sicca syndrome. Ann Intern Med 1978;89:888–92. 10.7326/0003-4819-89-6-888 102228

[15] Liang Y , Yang Z, Qin B, Zhong R. Primary Sjögren’s syndrome and malignancy risk: a systematic review and meta-analysis. Ann Rheum Dis 2014;73:1151–6. 10.1136/annrheumdis-2013-203305 23687261

[16] Rusinovich-Lovgach O , Plaza Z, Castro MF et al Cancer risk in Sjögren’s disease: a longitudinal cohort study on incidence, predictors, and mortality impact. Semin Arthritis Rheum 2025;73:152743. 10.1016/j.semarthrit.2025.152743 40349421

[17] Boyd JH , Randall SM, Ferrante AM et al Accuracy and completeness of patient pathways—the benefits of national data linkage in Australia. BMC Health Serv Res 2015;15:312. 10.1186/s12913-015-0981-2 PMC4529694 26253452

[18] Australian Institute of Health and Welfare. Aboriginal and Torres Strait Islander identification: Australian Institute of Health and Welfare; 2025. Available from: https://www.aihw.gov.au/reports-data/population-groups/indigenous-australians/indigenous-identification.

[19] World Health Organization. International statistical classification of diseases and related health problems, 10th revision: ICD-10. Geneva: World Health Organization, 2010.

[20] Grippo F , Frova L, Pappagallo M et al Beyond the underlying cause of death: an algorithm to study multi-morbidity at death. Popul Health Metr 2024;22:36. 10.1186/s12963-024-00356-8 PMC11653578 39696432

[21] Australian Bureau of Statistics. Data Explorer. Canberra; 2026. Available from: https://dataexplorer.abs.gov.au/.

[22] Australian Institute of Health and Welfare. General Record of Incidence of Mortality (GRIM) books: Australian Institute of Health and Welfare; 2025. Available from: https://www.aihw.gov.au/reports/life-expectancy-deaths/grim-books/contents/about.

[23] Lambert P. STPP: Stata module to compute Pohar-Perme non-parametric estimate of marginal relative (net) survival. Statistical Software Components. 2020; S458743.

[24] Dickman PW , Coviello E. Estimating and modeling relative survival. The Stata Journal 2015;15:186–215.

[25] Ederer FH. Instructions to IBM 650 programmers in processing survival computations. Bethesda, MD: National Cancer Institute, 1959.

[26] Lambert P. STPM3: Stata module to fit flexible parametric survival models: Boston College Department of Economics; 2024.

[27] Van Walraven C , Austin PC, Jennings A, Quan H, Forster AJ. A modification of the Elixhauser comorbidity measures into a point system for hospital death using administrative data. Med Care 2009;47:626–33. 10.1097/MLR.0b013e31819432e5 19433995

[28] Stagg V. Stata module to calculate Elixhauser index of comorbidity Statistical Software Components: Boston College Department of Economics.; 2015:S458077. Available from: https://ideas.repec.org/c/boc/bocode/s458077.html.

[29] Quan H , Sundararajan V, Halfon P et al Coding algorithms for defining comorbidities in ICD-9-CM and ICD-10 administrative data. Med Care 2005;43:1130–9. 10.1097/01.mlr.0000182534.19832.83 16224307

[30] Lee YH , Choi SJ, Ji JD, Song GG. Overall and cause-specific mortality in systemic lupus erythematosus: an updated meta-analysis. Lupus 2016;25:727–34. 10.1177/0961203315627202 26811368

[31] Rubio-Rivas M , Royo C, Simeón CP, Corbella X, Fonollosa V. Mortality and survival in systemic sclerosis: systematic review and meta-analysis. Semin Arthritis Rheum 2014;44:208–19. 10.1016/j.semarthrit.2014.05.010 24931517

[32] Lee YH , Song GG. All-cause and cause-specific mortality in rheumatoid arthritis: a meta-analysis. Z Rheumatol 2024;83:314–20. 10.1007/s00393-024-01538-3 38918258

[33] Almutairi KB , Inderjeeth CA, Preen DB, Keen HI, Nossent JC. Mortality trends among patients with rheumatoid arthritis in Western Australia. Rheumatol Ther 2023;10:1021–37. 10.1007/s40744-023-00562-0 PMC10326173 37335433

[34] Host LV , Lopez D, Keen HI et al Long-term epidemiology of systemic sclerosis in Western Australia: a population-level linked data study. J Rheumatol 2026;53:54–61. 10.3899/jrheum.2025-0525 41033823

[35] Nossent JC , Keen HI, Preen DB, Inderjeeth CA. Population-wide long-term study of incidence, renal failure, and mortality rates for lupus nephritis. Int J Rheum Dis 2024;27:e15079. 10.1111/1756-185X.15079 38396352

[36] Beltai A , Barnetche T, Daien C et al Cardiovascular morbidity and mortality in primary Sjögren’s syndrome: a systematic review and meta-analysis. Arthritis Care Res (Hoboken) 2020;72:131–9. 10.1002/acr.23821 30570824

[37] Lee AS , Scofield RH, Hammitt KM et al; Consensus Expert Panel (CEP) Members. Consensus guidelines for evaluation and management of pulmonary disease in Sjögren’s. Chest 2021;159:683–98. 10.1016/j.chest.2020.10.011 PMC8438162 33075377

[38] Zhong H , Liu S, Wang Y et al Primary Sjögren’s syndrome is associated with increased risk of malignancies besides lymphoma: a systematic review and meta-analysis. Autoimmun Rev 2022;21:103084. 10.1016/j.autrev.2022.103084 35341972

[39] Goulabchand R , Makinson A, Morel J et al Hospitalizations for infections in primary Sjögren’s syndrome patients: a nationwide incidence study. Ann Med 2022;54:2672–80. 10.1080/07853890.2022.2126517 PMC9542339 36173925

[40] Chastain DB , Spradlin M, Ahmad H, Henao-Martínez AF. Unintended consequences: risk of opportunistic infections associated with long-term Glucocorticoid therapies in adults. Clin Infect Dis 2024;78:e37–e56. 10.1093/cid/ciad474 37669916

[41] Eades LE , Hoi AY, Liddle R et al Systemic lupus erythematosus in Aboriginal and Torres Strait Islander peoples in Australia: addressing disparities and barriers to optimising patient care. Lancet Rheumatol 2024;6:e713–e26. 10.1016/S2665-9913(24)00095-X 38971169

[42] Nossent JC , Keen HI, Preen DB, Inderjeeth CA. Epidemiology and complications of rheumatoid arthritis in the indigenous Australian population. Rheumatol Int 2025;45:185. 10.1007/s00296-025-05951-y PMC12331860 40773135

[43] Singh AG , Singh S, Matteson EL. Rate, risk factors and causes of mortality in patients with Sjögren’s syndrome: a systematic review and meta-analysis of cohort studies. Rheumatology (Oxford) 2016;55:450–60. 10.1093/rheumatology/kev354 PMC5009445 26412810

[44] Sagy I , Cohen Y, Nahum Y et al Lower socioeconomic status worsens outcome of patients with systemic lupus erythematosus independently of access to healthcare. Lupus 2022;31:532–40. 10.1177/09612033221084518 35341361

