# Supplementary material for: Mortality and causes of death in patients with Sjögren disease in Western Australia: a population-based study
Source: Rheumatology (Oxford). 2026 Sep 10;65(10):keag496. doi: 10.1093/rheumatology/keag496 (PMC13653333; doi:10.1093/rheumatology/keag496)
Supplement: Supplementary file 1 [file keag496_supplementary_data.docx]

Supplementary material

**Supplementary Table S1: International Classification of Diseases Codes used for identifying patients with primary and associated Sjögren’s disease.**

|  |  | ICD-9/ICD-9-CM | ICD-10-AM |
| --- | --- | --- | --- |
| *Date range utilised* |  | *1980 – 1999* | *1999 – 2014* |
| Sicca syndrome (Sjögren's disease) |  | 710.2 | M35.0 |
| Other connective tissue disease (CTD) | Systemic lupus erythematosus | 710.0 | M32 |
|  | Systemic sclerosis | 710.1 | M34 |
|  | Dermatomyositis | 710.3 | M33.1 |
|  | Polymyositis | 710.4 | M33.2 |
| Rheumatoid arthritis |  | 714.0 | M05 - M06.0 |

The Hospital Morbidity Data Collection (HMDC) used ICD-9-CM until 1999. Subsequently, ICD-10-AM was used for diagnosis classification.

CTD: connective tissue disease, ICD-10-AM: International Classification of Diseases Codes, Tenth Revision Australian Modification, ICD-9: International Classification of Diseases, Ninth Revision, ICD-9-CM: International Classification of Diseases Codes, Ninth Revision, Clinical Modification.

**Supplementary Table S2: International Classification of Diseases cause of death analysis codes.**

| ICD Chapter | ICD-9-CM | ICD-10 |
| --- | --- | --- |
| Chapter I: Certain Infectious and Parasitic Diseases | 001-139 | A00-B99 |
| Chapter II: Neoplasms | 140-239 | C00-D48 |
| *Malignant neoplasms (excluding lymphoid, hematopoietic)* | 140-199 | C00-C80, C97 |
| *Lung cancer* | 162 | C33-C34 |
| *Lymphoid, haematopoietic malignancies* | 200-208 | C81-C96 |
| Chapter III: Diseases of the blood and blood-forming organs & certain disorders involving the immune mechanism | 280-289 | D50-D89 |
| Chapter IX: Diseases of the circulatory system | 390-459 | I00-I99 |
| *Hypertensive disease* | 401-405 | I10-I15 |
| *Ischaemic heart disease* | 410-414 | I20-I25 |
| *Other (cardiomyopathies etc, dysrhythmias)* | 420-427 | I30-I48 |
| *Heart failure (excludes that due to hypertension)* | 428 | I50 |
| *Cerebrovascular disease* | 430-438 | I60-I69 |
| *Pulmonary heart disease (including pulmonary embolism)* | I26-I28 | 415-417 |
| Chapter X: Diseases of the Respiratory System | 460-519 | J00-J99 |
| *Respiratory Infections* | 460-466, 470-478, 480-488 | J00-J06 J09-J18 J20-J22 |
| *Chronic lower respiratory disease* | 490-496 | J40-J47 |
| *Bronchiectasis* | 494-495 | J47 |
| *Interstitial Lung Disease* | 515-516, 717.2 517.8, 714.81 | J80-J99.0, J99.1, J99.8 |
| Other: |  |  |
| *Diabetes* | 250 | E10-E14 |
| *Dementia* | 290, 294.1, 331.2 | F00-F03, F05.1, G30, G31.1 |
| *Liver disease* | 070.22, 070.23, 070.32, 070.33,  070.44, 070.54, 070.6, 070.9,  456.0-456.2, 570-,571, 572.2-572.8, 573.3-573.4,  573.8-573.9 V42.7 | B18, I85, I86.4, I98.2, K70, K71.1, K71.3-K71.5, K71.7, K72-K74, K76.0, K76.2-K76.9, Z94.4 |
| *Renal disease* | 403.11, 403.91, 404.02-404.03, 404.12-404.13, 404.92-404.93, 582, 583.0-583.7, 585-586, 588,0 V56, V42.0, V45.1 | I12.0, I13.1, N03.2-N03.7, N05.2-N05.7, N18-N19, N25.0, Z49.0-Z49,2, Z94.0, Z99.2 |
| *Rheumatic Disease* | 446.5, 710.0-710.4, 714.0-714.2, 714.8, 725 | M05, M06, M31.5, M32-M34, M35.0, M35.1, M35.3, M36.0 |

ICD-10-AM: International Classification of Diseases Codes, Tenth Revision, ICD-9-CM: International Classification of Diseases Codes, Ninth Revision, Clinical Modification.

**Supplementary Table S3: Marginal Relative Survival during follow-up in SjD cohorts and age- and sex-matched controls at 5, 15 and 25 years of follow-up compared to Western Australian population mortality rates.**

| Group | Follow-up (years) | *N* at risk | Relative Survival (95% CI) |
| --- | --- | --- | --- |
| Control | 5 | 2974 | 1.01 (1.00, 1.01) |
|  | 15 | 1500 | 1.02 (1.01, 1.04) |
|  | 25 | 240 | 0.99 (0.95, 1.04) |
| Primary SjD | 5 | 301 | 0.90 (0.87, 0.94) |
|  | 15 | 128 | 0.88 (0.83, 0.93) |
|  | 25 | 13 | 0.77 (0.63, 0.94) |
| SjD + RA | 5 | 160 | 0.91 (0.83, 0.98) |
|  | 15 | 76 | 0.72 (0.59, 0.89) |
|  | 25 | 17 | 0.68 (0.55, 0.85) |
| SjD + CTD | 5 | 56 | 0.91 (0.85, 0.97) |
|  | 15 | 32 | 0.82 (0.74, 0.91) |
|  | 25 | ≤5 | 0.57 (0.36, 0.91) |

*Age standardized to the Australian 2001 Standard Population.*

CI: confidence interval, CTD: connective tissue disease, RA: rheumatoid arthritis, SjD: Sjögren Disease

**Supplementary Table S4: Mortality hazard ratios (HR) in SjD patients compared to matched controls, with and without the inclusion of sex and Elixhauser comorbidities as covariates (Cox regression).**

| **Group** | **Model 1**^2^ | | **Model** ^3^ | | **Model 3**^4^ | |
| --- | --- | --- | --- | --- | --- | --- |
|  | **HR (95% CI)** | ***p*-value** | **HR (95% CI)** | ***p*-value** | **HR (95% CI)** | ***p*-value** |
| **Controls** | 1 |  | 1 |  |  |  |
| ***Primary SjD*** | 1.8 (1.5, 2.0) | **< 0.001** | 1.7 (1.5, 2.0) | **< 0.001** | 1.5 (1.3, 1.7) | **<0.001** |
| ***SjD + RA*** | 2.1 (1.8, 2.5) | **< 0.001** | 2.2 (1.8, 2.6) | **< 0.001** | 1.8 (1.5, 2.1) | **<0.001** |
| ***SjD + CTD*** | 3.1 (2.2, 4.3) | **< 0.001** | 3.0 (2.2, 4.2) | **< 0.001** | 2.2 (1.6, 3.0) | **<0.001** |
| **Females** | NA |  | 1 |  | 1 |  |
| ***Males*** | NA |  | 1.5 (1.3, 1.7) | **< 0.001** | 1.4 (1.3, 1.6) | **<0.001** |
| **Elixhauser Comorbidities**^1^ | NA |  | NA |  | 1.11 (1.09, 1.13) | **<0.001** |

^1^The mean cumulative number of Elixhauser comorbidities by the final hospital admission, adjusted for age and sex.

^2^ Model 1: unadjusted hazard ratios.

^3^ Model 2: adjusted for sex as a covariate.

^4^ Model 3: adjusted for sex and Elixhauser Comorbidity number as a covariate.

CI: confidence interval, CTD: connective tissue disease, HR: hazard ratio, RA: rheumatoid arthritis, SjD: Sjögren Disease

**Supplementary Figure S1: Time-varying mortality hazard rates (Royston-Parmar model) and mortality hazard ratios (HR) with 95% confidence intervals for Sjögren disease cohorts and controls.**

**A:** Time-varying mortality hazard rates for SjD cohorts and controls. **B:** Time-varying mortality hazard ratios (HR) for SjD cohorts and controls.

Panel B: Shaded areas on Panel B represent 95% confidence intervals.

CTD: connective tissue disease, HR: hazard ratio, RA: rheumatoid arthritis, SjD: Sjögren disease
